# Biodiversity in a Cool-Climate Vineyard: A Case Study from Quebec

**DOI:** 10.3390/insects12080750

**Published:** 2021-08-19

**Authors:** Charles Vincent, Jacques Lasnier, Charles-Henri de Coussergues, Alain Baril

**Affiliations:** 1 Saint-Jean-Sur-Richelieu Research and Development Center, Agriculture and Agri-Food Canada, 430 Gouin Blvd., Saint-Jean-Sur-Richelieu, QC J3B 3E6, Canada; ch20100@yahoo.ca; 2 Co-Lab R&D Division d’Ag-Cord Inc., 655 Delorme St., Granby, QC J2J 2H4, Canada; 3 Vignoble de L’Orpailleur, 1086 Bruce St., Dunham, QC J0E 1M0, Canada; charles-henri@orpailleur.ca; 4 Plant Select, 1190 Principale West St., St.-Paul-d’Abbotsford, QC J0E 1A0, Canada; alainbaril@plantselect.ca

**Keywords:** arthropod, invasive insect species, cool-climate viticulture, grapevine, nectariferous plants, sustainability, agro-ecology

## Abstract

**Simple Summary:**

This paper documents research activities related to the biodiversity of the l’Orpailleur vineyard located in Dunham (Quebec, Canada) from 1997 to 2021. In a first phase starting in 1997, the biodiversity of insecticide-free and insecticide-treated parts of the vineyard was determined for several taxa. In a second phase starting 2004, entomological problems were addressed on an *ad hoc* basis as they unfolded. For example, at the request of viticulturists, research was conducted on the tarnished plant bug (*Lygus lineolaris*-Miridae) and on the system phytoplasmas/cicadellids/grapevines. In a third phase starting in 2014, management of plants between grapevine rows and areas adjacent to the vineyard was carried out to increase biodiversity with the aim to achieve arthropod control with minimal insecticide and acaricide use. To address the advent of a new pest, such as the Japanese beetle (*Popillia japonica*-Scarabaeidae), a biocontrol program based on the parasite *Istocheta aldrichi* (Tachinidae) was initiated.

**Abstract:**

In Quebec (Canada), viticulture has experienced steady growth in the last 35 years in terms of surfaces cultivated and value, although it is practiced in climatic conditions at the edge of what is considered a cool-climate area. This case study documents biodiversity studies conducted at the l’Orpailleur vineyard (Dunham, QC, Canada) from 1997 to 2021. In a first phase starting in 1997, the biodiversity of insecticide-free and insecticide-treated plots was determined for the taxa Scarabaeidae, Curculionidae, Chrysomelidae, Cicadellidae, Acari and Aranae. This step provided a baseline allowing to identify key arthropods. In a second phase starting in 2004, entomological issues were addressed on an *ad hoc* basis. In 2014, a third phase began with a perspective of sustainability and management of plant diversity in the vineyard to conserve natural enemies. Because of increased Japanese beetle (*Popillia japonica*-Scarabaeidae) populations and threats to vineyards, a biocontrol program based on the parasitoid *Istocheta aldrichi* (Tachinidae) was initiated. The unusually fast development of grapevines during the growing season, selection of flowering species, as well as selected arthropods associated with these flowering species, will be illustrated. Periodic update of protection programs will be required to address future challenges associated with climate change scenarios and world trade.

## 1. Introduction

Since antiquity, grape cultivation (i.e., viticulture) and wine making (i.e., oenology) have been practiced in regions near the Mediterranean Sea and in Transcaucasia where grape originated (*Vitis vinifera*- Vitaceae) [1,2]. From the antiquity to the mid-70’s, grape and wine production was mostly led by European countries. Since then, viticulture and oenology experienced worldwide robust and steady growth in terms of surfaces under cultivation and dollar value [3]. New regions, notably North America (USA, Canada), South America (Argentina, Chile), South Africa, Australia, New Zealand and, more recently, China, developed their viticultural and oenological industries. As of 2019, table wine and dried grapes were cultivated on 7.4 million ha worldwide [4]. About 50% of grape-cultivated areas are found in Spain, China, France, Italy, and Turkey. Meanwhile, the number of wine consumers have rapidly increased, as well as their eagerness for more quality wine. 

In various parts of the world, for example in California [5], viticulture has been mostly practiced as a monoculture until the 1990s. However, several factors have since been driving viticulture towards more sustainable practices [3], notably: (1) worldwide trade and competition, (2) climate change that may cause water shortage in some major grape-producing areas (e.g., [6,7,8]), (3) climate change that may exacerbate invasive species that challenge existing protection programs, and (4) consumer appeal for products based on sustainable practices. 

Sustainability is a concept that can be considered in several ways depending on region, agronomic situation, and market [9,10]. Some viticulturists embrace the sustainability concept to differentiate their organization because consumers see increased value in the way vineyards are managed. This approach can be based on economic (decrease costs), marketing (enhanced value perceived by consumers) or environmental (less pesticide input) considerations [9].

From an agroecology perspective, the idea of managing biodiversity to enhance ecological services has recently arisen [11,12,13,14]. In vineyards, the management of plant biodiversity can provide services and disservices affecting several agronomic parameters, notably soil fertility, health, carbon and N balance (input vs. uptake), soil erosion, competition with weeds, water management, and grape yield reduction [15]. 

Plant biodiversity can also impact arthropod biodiversity of vineyards, as documented in the last decade in several European studies. For example, ground cover plant management (i.e., Sweet Alyssum, Phacelia, Buckwheat, Faba Bean, Vetch and Oat) in vineyards of Northern Italy significantly affected the arthropod fauna, including beneficial groups providing ecosystem services, such as biological control of arthropod pests [16]. Phytoseiid predatory mites (*Typhlodromus pyri, Kampimodromus aberrans, Paraseiulus soleiger, Euseius finlandicus*) were shown to be more abundant on leaves of the vineyard plots with ground covers than in the control. Also in Northern Italy, the abundance of key natural enemies, notably predatory mites (*K. aberrans*, *Amblyseius andersoni*, *T. pyri*, *Phytoseius finitimus*), parasitic wasps, spiders, and some grapevine leafhoppers (*Zygina rhamni*) increased when the frequency of grass mowing was reduced [17].

In France, the abundance of six pest species, including *Panonychus ulmi* (Tetranychidae) and *Scaphoideus titanus* (Cicadellidae), and the beneficial mite *Orthotydeus lambi* (Tydeidae), was lower in vineyards with cover crops, while the abundance of the predatory mite *T. pyri* (Phytoseiidae) was higher in plots with cover crops [18]. Cover crops did not impact the intensity of diseases (*Plasmopara viticola*, *Uncinula necator* and *Guignardia bidwellii*).

In Austria, a study conducted in 32 organic and integrated vineyards concluded that predatory mites (*T. pyri* = 98.7%; *Euseius finlandicus* = 0.65%; *Paraseiulus talbii* = 0.62%) benefited from spontaneous vegetation cover in vineyard inter-rows and less intensive pesticide use [19].

In Romania, a study in 16 vineyards concluded that management intensity affected the diversity of some plant species and some invertebrate groups, but found the overall effect to be ambiguous [20].

In California, management of resident vegetation inside the vineyards and vegetation surrounding the vineyards is considered as complementary measures [5]. Summer cover crops grown inside vineyards substantially enhanced biological control of leafhoppers (*Erythroneura elegantula*) and thrips (*Franklinella occidentalis*) [5]. 

**Figure 1:**
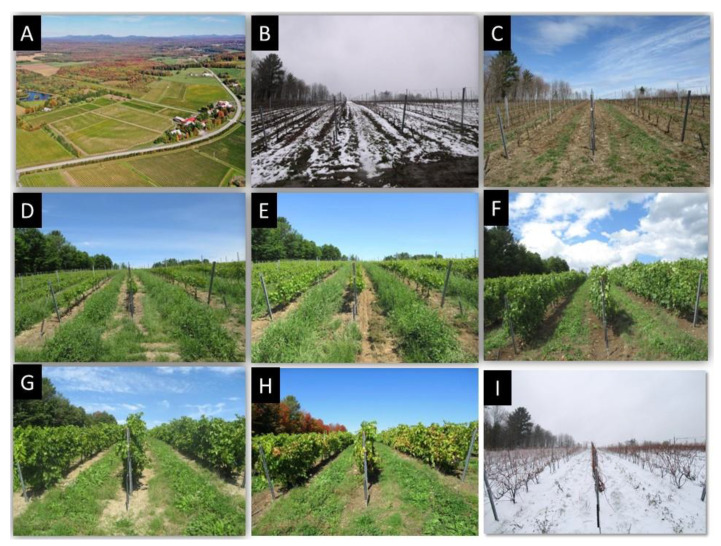
(**A**) Aerial view of the l’Orpailleur vineyard (Dunham, QC, Canada) and surrounding landscape. From (**B**–**I**): Development of vegetation deliberately established between rows of vines at the Bellevue site of the l’Orpailleur vineyard. (**B**) 11 April 2020; (**C**) 30 April 2020; (**D**) 30 May 2020; (**E**) 30 June 2020; (**F**) 31 July 2020; (**G**) 31 August 2020; (**H**) 30 September 2020; (**I**) 2 November 2020.

There is a paucity of publications on biodiversity of arthropods associated with vineyards conducted in cool-climate conditions, as well as on deliberate use of plants to manage these arthropod pests. This case study focuses on arthropod and plant biodiversity at the l’Orpailleur vineyard (Figure 1A). Located in Dunham (45°07′05″ N–72°49′16″ W), Quebec, Canada, l’Orpailleur is a vineyard where we conducted studies from 1997 to 2021 in three phases. In a first phase (1997–2003), briefly reviewed here, studies allowed for documentation of arthropod biodiversity and to identify key pests. In a second phase (2004–2013), entomological problems posed by established species were addressed as they arose. In a third phase (2014–2021), management of established arthropod pests was attempted by increasing biodiversity of vegetation in a context where the growing season is short and winters are very cold. The advent of invasive insects was addressed on a per case basis. 

**Viticulture in Quebec.** In Quebec, grape cultivation is practiced in meteorological conditions at the edge of cool-climate viticulture such that winter survival of grapevines is a major limiting factor. In winter, air temperatures frequently reach −30 °C and, occasionally, −35 °C. Grape cultivars may be assigned to three categories of rusticity: rustic cultivars can withstand −25 °C to −30 °C; semi-rustic cultivars, −20° to −24 °C; and sensitive cultivars, −17 °C to −19 °C. Rustic cultivars do not need winter protection. To protect semi-rustic and sensitive cultivars, some Quebec viticulturists hill vines with soil in late fall and unhill them in spring, or cover vines with geotextiles in the fall (see Figure 1 in [21]). Vine parts under snow cover are protected from harsh low temperatures. For example, in Frelighsburg, QC, when air temperatures hovered around −22 °C for several days in winter, soil temperatures at 3 cm seldom reached −5 °C [22]. The most striking method to cope with harsh winter conditions has been the use of geothermal energy (thanks to 15,000 m of tubes buried 2 m in the soil) to manage temperatures of vines grown under tunnels, as practiced in a winery located in Dunham, QC [23].

The season of vegetation in Quebec is short and of variable duration. In 2020, there was snow on 11 April on the soil at the l’Orpailleur vineyard (Figure 1B) and, in the fall, on 2 November (Figure 1I). Depending on prevailing spring temperatures, the woolly bud stage (B-Baggiolini; 03 Eichhorn-Lorenz) of grapevines has been observed from 27 April in 2009 to 19 May in 2020 (Table 1) [24]. On average, from 2009–2020 134 days were required to progress from the woolly bud stage to the berry ripe stage (N 38), i.e., beginning of harvest. The shortest and longest period were respectively 110 (2020) and 162 days (2010) on cultivar Seyval blanc in Dunham, QC (Table 1). These cool-climate conditions allow the production of specialty wines, such as Icewine and Vendanges tardives. Overall, the demand for Quebec wines is much higher than current wine production (C-H. de Coussergues, pers. comm.).

Finally, the size of vineyards (in ha) matters. While l’Orpailleur vineyard totals 37 ha of non- contiguous plots in production, the average surface in production of leading vineyards in Quebec is 11 ha [25], such that the proximity between cultivated vines and other plant species allows movement of arthropods from one plant species to another, and from plants growing outside the vineyards, but immediately adjacent to the cultivated vines.

**Arthropod biodiversity in two insecticide-free vineyards.** Starting in 1997, research projects were conducted on the biodiversity of arthropods in insecticide-free plots of two vineyards located in southern Quebec, notably l’Orpailleur and the Vineyard Dietrich-Jooss located in Iberville (45°15′26″ N–73°9′30″ W), Qc. This led to a number of scientific publications on the biodiversity of phytophagous arthropods [22,26,27,28]; Carabidae (124 species in Quebec vineyards); Cicadellidae (110 species [26,29]); Aranae (97 species); Curculionidae (73 species); Coccinellidae (22 species [30]); and Chrysomelidae (59 species). As stated by Kreiter [31], these publications, reviewed by Vincent et al. [28], constitute baselines that are unique contributions to the study of arthropod diversity in minimally managed vineyards. Thanks to the first phase, we identified key arthropods of vineyards in Southern Quebec.

As Dietrich-Jooss vineyard phased out its operations in 2004, we focused our research efforts on the l’Orpailleur vineyard. As the Quebec viticultural industry developed and the research studies unfolded, specific arthropod issues, such as the tarnished plant bug (*Lygus lineolaris*- Miridae), were addressed on an *ad hoc* basis [32,33]. *L. lineolaris* feeds on ca. 398 host plants and is highly mobile in vineyards [33]. Hence, it is a pest of great concern. Meanwhile, studies on the arthropod fauna associated with floral strips cultivated between rows of apple trees were conducted at the experimental farm of Agriculture and Agri-Food Canada located at Frelighsburg, QC [34]. They demonstrated the attractiveness of floral strips (*Tanacetum vulgare*, *Chrysanthemum maximum*, *Aster tongolensis*, *Achillea millefolium;* all Asteraceae) towards natural enemies, notably hymenopterans (Ichneumonoidea, Chalcidoidae, and Proctotrupoidea) and dipterans (Cynipoidea, Syrphidae, and Tachinidae) species [35].

**Managing plant and arthropod biodiversity in Quebec vineyards.** Before the advent of cultivated vines in the 17th century, there were wild vines in Quebec [36]. False Virginia creeper (*Parthenocissus vitacea*) and riverbank grape (*Vitis riparia) are* vine species *native* to Quebec, and Virginia creeper (*Parthenocissus quinquefolia)* is an introduced species [37]. If these plant species are left unmanaged in areas adjacent to the vineyards, they may harbor arthropod pests of cultivated vines as well as beneficials. 

As viticulture developed in Quebec, preventive and sustainable methods to manage most arthropod pests (Table 2 in [38]; Table 1 in [21]) were increasingly needed. To that end, innovative approaches have been documented in other grape-producing areas, notably in Australasian [39] and Californian [40] vineyards. In these examples, several ecosystem services (e.g., biodiversity conservation, biological control, nutrient management, erosion control, weed suppression) were enhanced by managing the floor vegetation between and on vine rows. In New Zealand, buckwheat (*Fagopyrum esculentum*- Polygonaceae) sown between vine rows enhanced several services, notably providing nectar resources to natural enemies. Wilson and Daane [41] reported that planting flowering cover crops (i.e., buckwheat (*F. esculentum*), sweet alyssum (*Lobularia maritima*), purple tansy (*Phacelia tanacetifolia*), and clovers (*Trifolium* spp.) in summer is relatively rare in California, primarily because these plants compete with water, which is a limiting resource. This conclusion is irrelevant for Quebec vineyards, where water is not a limiting factor because of abundant rainfall throughout the growing season. Clearly, the management of plant diversity must be tailored for a given viticultural region: different terroirs mean different biodiversities.

**Figure 2:**
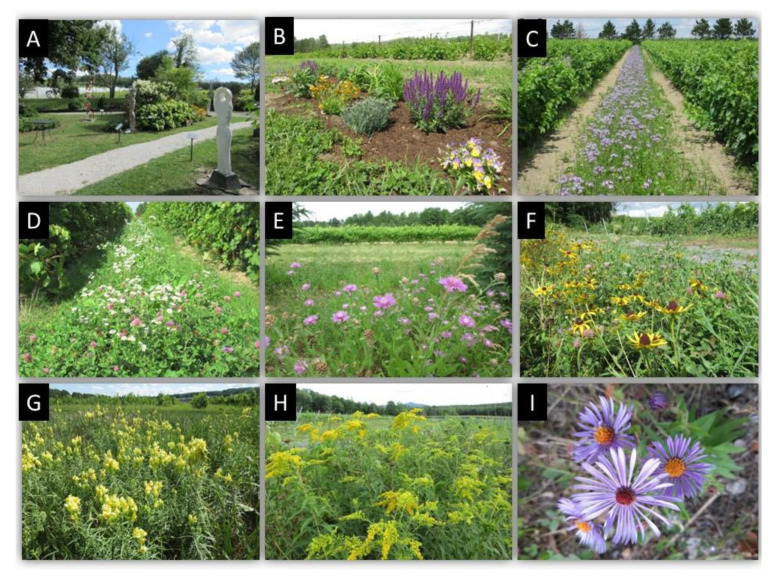
(**A**) Sculpture and vegetation established between the buildings of the winery and the vineyard (in background) for aesthetic purpose (Dunham, QC); (**B**) Vegetation established in the vineyard (in background) for aesthetic purpose; (**C**) *Phacelia sp*. (in flower) planted between vine rows to increase floral resources. Destroyed by hilling in the fall, they are re-sown in spring; (**D**) Wild *Aster sp*., *Centaurea sp*. and *Trifolium sp*. plants established for several years between vine rows. These plants are mown twice per season; (**E**) Wild flowers *Centaurea vochinensis* (short-fringed knapweed); (**F**) Wild *Rudbeckia latiniata* (cut-leaved coneflower); (**G**) *Linaria vulgaris* (yellow toadflax); (**H**) *Solidago canadensis* (Canada goldenrod); (**I**) *Aster patens* (late purple aster). See Table 2A for flowering times of (**E**–**I**).

By favouring a diversity of spontaneous or sown plant species in and areas adjacent to cultivated vines (Figure 2A–I), we aimed to increase nectar resources and refuges, and thereby populations of natural enemies throughout the season. The choice of winter protection of vines may influence cover vegetation and, thus, phytosanitary practices. In semi-rustic and vinifera cultivars covered with geotextiles and uncovered rustic cultivars, establishment of soil cover by spontaneous vegetation, i.e., native or non-native plants issued from seed banks and rhizomes in the soil, was achieved. However, hilling/unhilling grapevines precluded the establishment of a permanent cover vegetation on and between grapevine rows. 

Other considerations for managing plant biodiversity in cool-climate viticultural situations were: (1) the selected sown plants must have a rapid growth and provide adequate and sustained ecological services for several natural enemies present at different moments in the season. For example, nectariferous plants must bloom for a significant duration such that insects relying on nectar supply will be retained for a long period; (2) the flowering plants must be relatively inexpensive; (3) spontaneous flowering plants should remain established for several years; and (4) from an entomological point of view, the chosen plant species must not be a preferred host of a potential arthropod pest species. For example, the spotted wing drosophila (*Drosophila suzukii*-Drosophilidae), first identified in 2012 in Quebec vineyards [42], do not presently represent a threat to vineyards as grapes are not its preferred host. Enhancement of pollination services, a consideration often mentioned in scientific publications [43], is irrelevant in vineyards as entomophilous pollination is not required for grape production [2].

## 2. Materials and Methods

At the l’Orpailleur vineyard, two methods to mitigate winter damage to vines were implemented; with one method, plots where vines were hilled with soil in the fall were unhilled in spring (about 56% of surfaces at l’Orpailleur). This implied that the soil between vine rows was devoid of cover crops. However, spontaneous (i.e., native or non-native plants that emerged from seed bank or other means like rhizomes) plants can grow in surfaces adjacent to these plots. Annual plants (e.g., *Phacelia* sp.) were sown between vine rows. Over the years, we selected several plant species that were sown on a trial-and-error basis on of surfaces of hilled/unhilled vine plots. One important consideration was that the routing system of these plants should not interfere with hilling/unhilling operations. In order to mitigate winter damage, with the second method, vines were covered with geotextiles in the fall and removed in spring (about 18% of surfaces). This allowed spontaneous vegetation to grow between vine rows or outside and areas adjacent to these plots. At the Bellevue site (45°07′05″ N–72°49′16″ W) located ca. 3.2 km from the main vineyard/winery, plots with rustic vines (e.g., Frontenac blanc, Frontenac rouge, *Musca oceola*) were left unprotected for winter (about 26% of surfaces). 

Meteorological data was gathered from Agrométéo Québec [44] as described in the Appendix A. 

From 2014 to 2020, all plots were visited at least twice per week from 1 April (Woolly bud- B 03) to 1 October (Leaf fall- O 43) to: (1) determine the phenological stages of the vines; 2) identify and visually estimate the abundance of spontaneous flowering plants and their flowering period between vine rows of hilled/unhilled plots (at random on 3 × 10 m strips), and non-hilled plots (at random on 5 × 10 m strips) outside the vineyard and immediately adjacent surfaces; and (3) determine the abundance of selected arthropod species on cover crops between rows and on spontaneous plants growing in areas adjacent to cultivated plots. From April to harvest, the arthropods and their damage were visually monitored weekly on at least 100 vine plants per plot by one of us (J. Lasnier). Based on our experience, two species needed to be specifically monitored with the following methods. 

At the woolly bud stage (B 03) (i.e., 25-day degrees >10 °C), 3 pheromone traps were positioned in a 6 ha Seyval block (hilled/unhilled plot) to monitor adult male grape berry moths (*Paralobesia viteana*- Tortricidae). The traps (Multipher III, Trécé Inc., Salinas, CA, USA) were visited twice per week to determine the capture of male moths. The pheromone dispensers were changed every third week. Male moths were caught from the green shoot stage (D 06) until leaf fall (O 43), which coincided with the first freeze in the fall. For example, first freeze occurred at 1400 day-degrees (>10 °C) on 11 October 2018; 1189 day-degrees (>10 °C) on 31 October 2019, and 1297 day-degrees (>10 °C) on 8 October 2020. In the 6 ha plots where the traps were positioned, estimation of larval damage was carried out by random examination of 100 fruit clusters at the stage when berries were ripe (N 38) in 2018, 2019, and 2020. 

**Figure 3:**
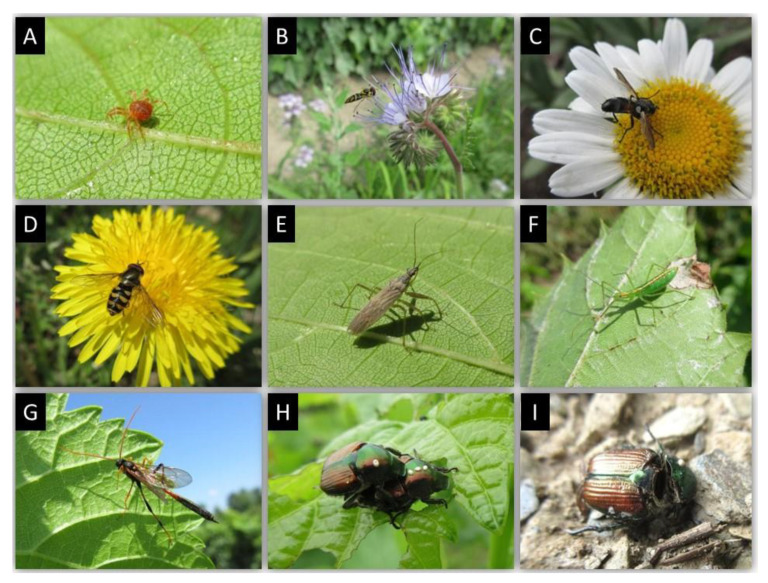
(**A**) *Anystis baccarum* (Anystidae) adult on vine leaf; (**B**) *Allograpta obliqua* (Syrphidae) adult feeding on a Phacelia flower; (**C**) *Cylindromyia binota* (Tachinidae) adult on a *Leucanthemum vulgare* flower; (**D**) *Megasyrphus sp.* (Syrphidae) adult feeding on a *Taraxacum officinale* (common dandelion) flower; (**E**) *Nabis americoferus* (Nabidae) adult under a grape leaf; (**F**) *Zelus luridus* (Reduviidae) nymph; (**G**) *Therion fuscipennis* (Ichneumonidae) adult; (**H**) *Istocheta aldrichi* (Tachinidae) eggs on pronotum of *Popilllia japonica* (Scarabaeidae) adult; (**I**) After devouring internal tissues of *Popilllia japonica* (Scarabaeidae) adult, *Istocheta aldrichi* (Tachinidae) overwinters as pupa in the dead carcass of its host.

In 2018, 2019, and 2020, the abundance of immatures and adults of the predator *Anystis baccarum* (Anystidae) (Figure 3A) was estimated twice per week by tapping 100 vine shoots, flower buds or fruit clusters over a 2 L plastic container. Specimens that fell into the container were tallied as *Anystis baccarum* immatures and adults on 100 shoots, flower buds or fruit clusters. 

## 3. Results and Discussion

The most common species of spontaneous flowering plants belonged to 29 genera (12 families) (Table 2A). In vineyards that were hilled/unhilled, four species of annual plants belonging to three families were sown (Table 2B), and spontaneous flowers strips established immediately adjacent to the vineyards were left unmowed or were mowed in late fall. 

**Figure 4:**
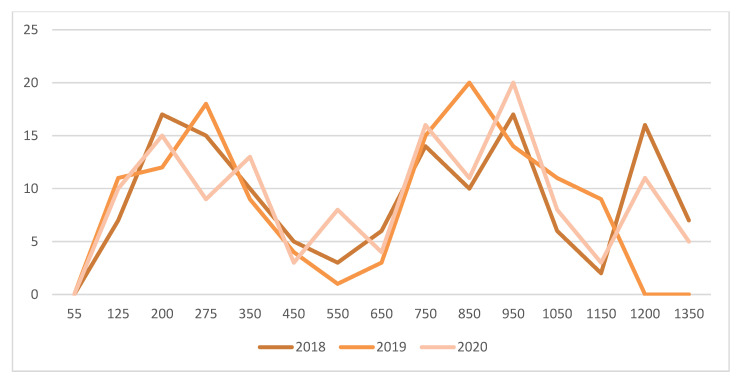
Total captures of adult male grape berry moths (*Paralobesia viteana)* by 3 pheromone traps positioned in a 6 ha Seyval plot at l’Orpailleur vineyard in 2018, 2019 and 2020. Horizontal axis are day degrees (>10 °C) starting at the green-shoot stage (D 06).

A first mowing of spontaneous plants established between vine rows was conducted two weeks after the first peak captures of adult male grape berry moths (*Paralobesia viteana*- Tortricidae) with pheromone traps, ca. 450 day-degrees (>10 °C) (Figure 4). This allowed generalist predators and adults of braconid, tachinid, and ichneumonid parasites to feed on floral resources. Several parasitoids of lepidopteran larvae, such as *Therion fuscipennis* (Ichneumonidae) (Figure 3G), were found on flower strips. A second mowing of plants between vine rows was conducted at the end of August (after the second peak of captures of adult male grape berry moths), thus providing time for parasitism to effectively occur. Egg parasitism of grape berry moth by *Trichogramma* spp., as reported in Pennsylvania by Nagarkatti et al. [45], was also found. At the stage when berries were ripe (N 38), there were respectively 2, 0, and 1 larva/100 clusters examined in 2018, 2019, and 2020. These low population levels did not warrant insecticidal treatments.

**Figure 5:**
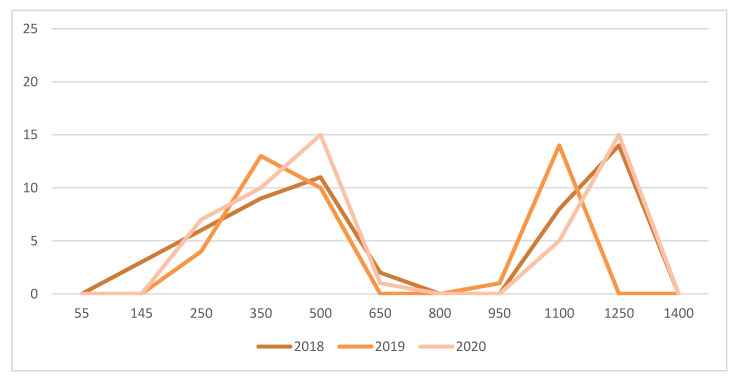
Abundance of *Anystis baccarum* immatures and adults on 100 fruit buds/clusters in 2018, 2019, and 2020. Horizontal axis are day degrees (>10 °C) starting at the green-shoot stage (D 06).

Amongst the 60 cicadellid species found in Quebec vineyards [26,29], the most common were *Empoasca fabae*, *Erythroneura comes*, *Erythroneura tricincta*, *Erythroneura ziczac* and *Erythroneura vitis*. The predatory mite *Anystis baccarum* (Anystidae) was an efficient predator of nymphs and adult leafhopper. It has two generations per season in Quebec. Nymphs were found at the beginning of June (Figure 5). The first generation ended by mid-July. The peak of the second generation occurred at the end of August. To foster predation of cicadellids by immatures and adults of the two generations of *A. baccarum*, flower strips were maintained between vine rows and areas adjacent to cultivated vines. 

The flower strips also conserved other predators and parasites (Table 3). For example, the chrysopids *Chrysopa carnea* and *Hemerobius humilinus*; the coccinellids *Coccinella septempunctata*, *Coleomegilla maculata*, *Harmonia axyridis* and *Hippodamia* spp.; the nabid *Nabis* spp.; and the reduviid *Zelus* spp. (Figure 3F) were often observed predating on several stages of leafhoppers. We also observed egg parasites of leafhoppers, as reported in New York State by Williams and Martinson [46] and English-Loeb et al. [47].

Some phytophagous mirids and pentatomids were parasitized by the tachinid *Cylindromya binota* (Figure 3C), a species frequently found in flower strips. This species, reported by Normandin [48], could also be important for the biocontrol of the brown marmorated stink bug (*Halyomorpha halys- Pentatomidae)*. 

A highly polyphagous insect native to northern China, Japan, and Far East of Russia, the Japanese beetle (*Popillia japonica-* Scarabaeidae) was first discovered in North America in Riverton, New Jersey, in 1916 [49,50,51]. As of 1983, it was present on ornamental roses grown in Bedford, Qc, a locality near the Quebec/Vermont border [52]). Reported as a pest of New England vineyards [53], it has been present in vineyards of southern Quebec since 2010, its populations increasing ever since [54]. From 1920–1933, the United States Department of Agriculture (USDA) imported and released 49 species of natural enemies in Northeastern USA, including *Istocheta aldrichi,* a tachinid that parasitizes newly emerged adults [50]. Reported in Ontario by O’Hara [55], *I. aldrichi* was observed in 2014 associated with Japanese beetle adults in vineyards of Southern Quebec [54]. Following oviposition of their eggs on the pronotum of Japanese beetle adults (Figure 3H), *I. aldrichi* complete their larval development and pupate in their host, which mummifies before winter (Figure 3I). The following year, *I. aldrichi* adults emerge around two weeks before the appearance of Japanese beetle adults [54]. Before the availability of their host, *I. aldrichi* adults feed on nectar of flowering plants in the vineyards. In plots where a high proportion of Japanese beetle adults had of *I. aldrichi* eggs on their pronotum, natural control was effective and Japanese beetle populations and their damage were maintained at tolerable levels in 2018, 2019, and 2020 such that insecticidal treatment was not necessary.

**Other services provided by managing biodiversity in vineyards**. As argued by Garcia et al. [8], viticulturists generally pursue several objectives when deliberately increasing plant biodiversity. For example, in semi-arid vineyards of British Columbia, Canada, Vukicevich et al. [56,57] demonstrated that the management of cover plants can change root fungal communities and plant-soil feedback.

Currently, wine consumers are increasingly demanding wine produced with environmentally sustainable practices, as exemplified by Tompkins et al. [39] for New Zealand and de Coussergues [58] in Quebec. Enhancing plant diversity in vineyards addresses this demand.

Increasing diversity of plants can also be a way to enhance the aesthetic experience of agrotourists visiting wineries (Figure 2B). Some viticulturists of Southern Quebec further increased the aesthetic experience by organizing symposia of sculptures amongst ornamental plants growing adjacent to their vineyards (Figure 2A).

**Three phases of entomological research culminated in managing biodiversity**

A series of research projects that occurred over three phases allowed for the development of sustainable protection programs for Quebec vineyards. In a first phase beginning in 1997, assessment of the biodiversity of arthropods in insecticide-free plots as part of two commercial vineyards was carried out to establish baselines. Key arthropod pests and the risk they incurred was identified (see Table 2 in [59]; Table 1 in [21]). In a second phase beginning in 2004, arthropod problems were researched on an ad hoc basis, and we applied the main principles involved in Integrated Pest Management relevant to viticulture [60]: know your pests, favour natural enemies, monitor pests, and apply pesticides as a last resort. In this phase, research on the biodiversity of leafhoppers was critical to address a potential problem caused by phytoplasmas. In a third phase beginning in 2014, management of plant diversity in the vineyard and its adjacent surroundings has been adopted as a practice to conserve natural enemies with the aim to provide a broad-reaching control measure such that commercially acceptable yields of grapes would be achieved with low quantities of insecticides and acaricides per ha. In 1997, participating vineyards applied approximately five insecticide acaricide sprays per season. Since 2010, <1.5 insecticide sprays have been applied per season at l’Orpailleur. Since 2015, only one insecticide spray was applied before bloom (I 23), and none were applied from the time the fruit set (J 27) until harvest (N 38). This constitutes our entomological benchmark by which we appraise our overall success in terms of protection program, including the effect of managing plant biodiversity.

**Looking forward**

The current protection programs practiced in Quebec vineyards will likely be challenged by a number of factors. First, challenges incurred by invasive arthropods will need to be addressed as they come. The spotted wing drosophila (*Drosophila suzukii-*Drosophilidae) was found in 2012 in Quebec vineyards [42] and, at present, it is not a concern for viticulturist as grape is a non-preferred host. The brown marmorated stink bug (*Halyomorpha halys- Pentatomidae*) [61] has been reported in the Montreal area from 2008 to 2018 [62,63] but, as of 2020, it has not been reported as a pest of vineyards. The grape rootworm (*Fidia viticida*-Chrysomelidae), an insect native to North America hitherto restricted to Ontario in Canada, was found in 2016 associated with *Parthenocissus quinquefolia* and *Vitis riparia* in Laval, QC [64]. Absent before 2018, the grape plume moth (*Geina periscelidactylus-*Pterophoridae) was recently found in Quebec vineyards. Although these arthropods are a minor addition to the biodiversity of arthropods, their advent is a cause of concern because they have the potential to upset existing programs.

Some pests present in Southern Ontario, such as the blotch leafminer (*Antispila viticordifoliella-*Heliozelidae), the grape mealybug (*Pseudococcus maritimus-*Pseudococcidae), the European fruit lecanium scale (*Parthenolecanium corni-*Coccidae), the grape cane girdler (*Ampeloglypter ampelopsis-*Curculionidae), and the grape cane gallmaker (*Ampeloglypter sesostris-*Curculionidae), may expand their current geographical distribution under climate change scenarios. The spotted lanternfly (*Lycorma delicatula-*Fulgoridae) has been found for the first time in 2014 in Pennsylvania and, as of 2019, was present in several New England States [65]. It is polyphagous (>65 host plants), and *Vitis vinifera* is one of its preferred hosts. As of 2021, it was absent from Canada, and has been added to the list of regulated species by the Canadian Food Inspection Agency [66]. If present, this species is likely to cause some concerns to viticulturists and impact protection programs.

Second, grapevine diseases vectored by insects are increasingly causing concern to viticulturists. In 2006, Bois noir was first detected in Canada by Rott et al. [67] and was quickly eradicated. This prompted research on phytoplasmas associated with grapevines in Canada as well as their insect vectors, notably cicadellids [29,47,68]. Amongst the 37 leafhopper species that were phytoplasma DNA-positive, 11 are known vectors, the strongest potential vector being *Macrosteles quadrilineatus* (Cicadellidae). As of 2020, Flavescence dorée and Bois noir, two quarantine phytoplasmas associated with cultivated grapewines were absent from Canada.

From 2006 to 2008, a study about leafroll-associated virus and their vectors was conducted in the Finger Lakes region of New York State [69]. Grape mealybug (*Pseudococcus maritimus-*Pseudococcidae), European fruit lecanium (*Parthenolecanium corni-*Coccidae), and cottony maple scale (*Pulvinaria acericola-*Coccidae) were identified as vector species. In the 2010s, viral diseases associated with cultivated grapevines were increasingly reported. In surveys conducted in vineyards of British Columbia (Canada) in 2014 and 2015, Poojari et al. [70] found several viruses, the most prevalent being GLRaV-3 (16.7% of composite samples). Present at low levels, *Pseudococcus maritimus (*Pseudococcidae) and *Parthenolecanium corni (*Coccidae) were identified by barcoding. In 2018–2019 in Quebec vineyards, three viruses (i.e., grapevine rupestris stem pitting-associated virus, grapevine leafroll-associated virus (GLRaV) 3 and 2, and hop stunt viroid (HSVd)) largely dominated the virome of grapevines [71]. Currently, mealybugs and soft scales are at very low levels in Quebec vineyards and their management with plant diversity have yet to be researched to develop efficient and sustainable management methods for phytoplasmas and virus vectors. Biovigilance of established and newly established arthropods species is in order.

Third, the advent of climate change scenarios will directly impact the development all arthropods as they are poikilotherms, as well as cultivated and non-cultivated plants. Climate change is likely to shift the types of vines cultivated worldwide [72,73,74,75,76]. Based on a probabilistic model, Roy et al. [77] evoked several scenarios involving temperature increase such that *Vitis vinifera* will survive winters by 2040–2050 and new regions of Quebec will be amenable to viticulture. However, they point out uncertainties related to abundant rainfall and cloud cover that could negatively impact vine growth and grape production. All the plants in vineyards and areas adjacent to them will also be impacted. It is believed that being at the edge of cool-climate, Quebec viticulture will benefit from climate change scenarios. These scenarios overlook uncertainties related to extreme temperature variations in summer (i.e., variations in the duration of frost-free growing season) and winter (i.e., variation in below-freezing temperatures and snow cover in winter). Climate change will impact plants and arthropods unequally, creating more uncertainty for the whole viticultural industry. Consequently, an update of our current protection program, including management of arthropods with plant biodiversity, will have to be performed to cope with the prevailing situation.

## Figures and Tables

**Table 1:** Date of occurrence of phenological stages of grapevines (cultivar Seyval) grown at the l’Orpailleur vineyard (Dunham, QC) from 2009 to 2020.

Phenological Stage (Baggiolini Eichhorn-Lorenz) *	Day Degrees (>10 °C) Starting 1 April	2009	2010	2011	2012	2013	2014	2015	2016	2017	2018	2019	2020
Woolly bud (B 03)	25	27 April	5 April	2 May	2 May	30 April	9 May	4 May	12 May	27 April	3 May	10 May	19 May
Bud swell (C 05)	48	7 May	1 May	13 May	13 May	2 May	13 May	6 May	19 May	6 May	8 May	23 may	21 May
Green shoot (D 06)	56	9 May	3 May	18 May	15 May	4 May	14 May	8 May	21 May	13 May	10 May	25 may	23 May
2 to 3 leaves unfolded (E 09)	75	20 May	6 May	20 May	19 May	6 May	16 may	9 May	23 May	17 May	16 May	30 May	26 May
Inflorescences clearly visible (F 12)	125	6 June	23 May	28 May	24 May	11 May	29 May	18 May	28 May	28 May	26 May	10 June	29 May
Single flowers in compact groups (G 15)	145	11 June	25 May	30 May	26 May	20 May	02 June	24 May	30 May	31 May	29 May	13 June	4 June
Flowers separating (H 17)	170	14 June	27 May	1 June	29 May	23 May	04 June	27 May	1 June	8 June	31 May	17 June	6 June
50% Flowering (I 23)	315	30 June	19 June	21 June	19 June	19 June	23 June	19 June	21 June	23 June	21 June	2 July	22 June
50% Fruit set (J 27)	375	7 July	25 June	27 June	24 June	25 June	29 June	25 June	27 June	30 June	29 June	6 July	27 June
Veraison beginning (M 35)	900	29 August	11 August	13 August	10 August	12 August	21 August	17 August	14 August	21 August	08 August	21 August	09 August
Beginning of harvest (N 38)		22 September	15 September	13 September	11 September	16 September	15 September	15 September	13 September	18 September	9 September	17 September	7 September
Day Degrees (>10 °C) at beginning of harvest		1036	1211	1185	1217	1169	1107	1216	1211	1087	1249	1072	1184
**No. Days between Woolly bud (B 03) and beginning of harvest (N 38)**		147	162	133	131	138	128	133	123	143	128	129	110

* In parentheses, letters and numbers refer respectively to the Baggiolini and Eichhorn-Lorenz phenological systems [24].

**Table 2:** (**A**) Most common species of spontaneous flowering plants established in all plots between rows in the vineyard and in strips adjacent to the vineyard from April (Ap.) to October (Oc.). (**B**) Plants sown between rows of hilled/unhilled vines. In a given month, the intensity of the color is proportional to the abundance of floral resources.

Family	Latin Name (Figure No.)	# sp.	Common Name	Ap	Ma	Ju	Ju	Au	Se	Oc
**(A)**										
Apiaceae	*Daucus carota*		Wild carrot							
										
Apocynaceae	*Asclepias* spp.	3	Milkweed							
										
Asteraceae	*Achillea* spp.	2	Common yarrow							
	*Aster* spp. (2I)	3	Aster							
	*Centaurea* spp. (2D, 2E)	3	Knapweed							
	*Leucanthemum vulgare*		Ox-eye daisy							
	*Crepis capillaris*		Hawkweed							
	*Erigeron* spp.	3	Fleabane							
	*Inula helenium*		Elecampane							
	*Matricaria chamomilla*		Wild chamomile							
	*Rudbeckia* spp. (2F)	3	Coneflower, Black eyed							
	*Solidago* spp. (2H)	3	Goldenrod							
	*Sonchus asper*		Spiny-leaved thristle sow							
	*Tanacetum vulgare*		Common tansy							
	*Taraxacum officinale*		Common dandelion							
	*Tragopogon pratensis*		Meadow goatsbeard							
										
Brassicaceae	*Erysimum cheiranthoides*		Wormseed mustard							
	*Hesperis matronalis*		Dame’s violet							
										
Caryophyllaceae	*Lychnis alba*		White campion							
	*Silene* *cucubalus*		Blader campion							
	*Stellaria graminea*		Grass-leaved starwort							
										
Fabaceae	*Trifolium* spp. (2D)	2	Clover							
Lamiaceae	*Monarda didyma*		Oswego tea							
										
Onagraceae	*Oenothera* spp.	2	Primrose							
										
Plantaginaceae	*Linaria vulgaris* (2G)		Butter and eggs							
	*Veronica officinalis*		Common speedwell							
										
Polemoniaceae	*Phlox pilosa*		Downy phlox							
										
Ranunculaceae	*Ranunculus* spp.	4	Buttercup							
										
Rosaceae	*Potentilla* spp.	5	Cinquefoil							
**(B)**										
Brassicaceae	*Sinapis alba*		White mustard							
	*Brassica nigra*		Black mustard							
										
Hydrophyllaceae	*Phacelia tanacetifolia* (2C)		Lacy phacelia Purple Tansy							
										
Polygonaceae	*Fagopyrum esculentum*		Buckwheat							

**Table 3:** Most common beneficials found on vines and on spontaneous flowering species strips established between vines and areas adjacent to the vineyard. (Figure 2C,D) and adjacent to the vineyard (Figure 2E–I) at the l’Orpailleur vineyard, Dunham, QC. Prey: 1 = several species; 2 = phytophagous mites; 3 = leafhoppers; 4 = lepidopteran immatures; 5 = aphids; 6 = mealybugs; 7 = Pentatomidae, Miridae; 8 = *Popillia japonica*; x = minimally abundant and occasional; xx = moderately abundant and common; xxx = abundant and common; xxxx = very abundant and common.

Order	Family	Latin Name (Figure No.)	Prey	Abundance
Araneae	Araneidae	*Araneus diadematus*	1	xx
		*Araniella displicata*	1	xx
		*Argiope aurantia*	1	x
	Thomisidae	*Xysticus* spp.	1	x
Coleoptera	Coccinellidae	*Anatis* spp.	1	x
		*Coccinella septempunctata*	1	xx
		*Harmonia axyridis*	1	xxx
		*Hippodamia convergens*	1	xx
		*Coleomegilla maculata*	1	xxx
Diptera	Syrphidae	*Allograpta obliqua* (3B)	1	xx
		*Megasyrphus* spp. (3D)	1	xx
		*Ocyptamus fascipennis*	6	xx
		*Syrphus rectus*	1	xx
		*Toxomerus* spp.	1	xx
	Tachinidae	*Cylindromyia binota* (3C)	7	xx
		*Istocheta aldrichi* (3H-3I)	8	xxxx
		*Jurinia pompalis*	4	xx
Hemiptera	Nabidae	*Nabis americoferus* (3E)	1	xx
		*Nabis roseipennis*	1	xx
	Reduviidae	*Zelus luridus* (3F)	1	xx
Mesostigmata	Phytoseiidae	*Amblydromella* spp.	2	x
		*Neoseiulus fallacis*	2	x
Trombidiformes	Anystidae	*Anystis baccarum* (3A)	2.3	xxxx
	Trombidiidae	*Allothrombium lerouxii*	2,3,4	x
Hymenoptera	Braconidae	*Aleiodes* spp.	4	x
	Ichneumonidae	*Therion* spp. (3G)	4	xx
		*Ichneumon* spp.	4	xx
	Mymaridae	*Anagrus* spp.	3	x
	Trichogrammatidae	*Trichogramma* spp.	4	x
Neuroptera	Chrysopidae	*Chrysoperla carnea*	1	xx
		*Chrysopa oculata*	1	xx
	Hemerobiidae	*Hemerobius* spp.	1	xx

## Data Availability

Not applicable.

## Appendix A

As briefly referred to in Materials and Methods, meteorological data was gathered from Agrométéo Québec [44] with the following steps: Step 1- Click on section « Sommaire et prévision»; Step 2- Click on «Quotidien ?»; Step 3- Select a stating and ending date by clicking «Choisir une date» and «Fin du sommaire»; Step 4- Chose a base temperature by clicking «Choix de la T^O^ de base». We chose 10 °C; Step 5- Chose a station. We chose «Dunham » and saved it; Step 6- Generate a table according to the previously defined parameters by clicking «Obtenir sommaire».
